# A comparison study of different physical treatments on cartilage matrix derived porous scaffolds for tissue engineering applications

**DOI:** 10.1088/1468-6996/15/6/065001

**Published:** 2014-11-12

**Authors:** Ali Moradi, Sumit Pramanik, Forough Ataollahi, Alizan Abdul Khalil, Tunku Kamarul, Belinda Pingguan-Murphy

**Affiliations:** 1 Department of Biomedical Engineering, Faculty of Engineering Building, University of Malaya, 50603 Kuala Lumpur, Malaysia; 2 Department of Surgery, Faculty of Medicine Building, University of Malaya, 50603 Kuala Lumpur, Malaysia; 3 Department of Orthopaedic Surgery, Faculty of Medicine Building, University of Malaya, 50603 Kuala Lumpur, Malaysia

**Keywords:** cartilage matrix, porous scaffold, cross-linking, physical treatment

## Abstract

Native cartilage matrix derived (CMD) scaffolds from various animal and human sources have drawn attention in cartilage tissue engineering due to the demonstrable presence of bioactive components. Different chemical and physical treatments have been employed to enhance the micro-architecture of CMD scaffolds. In this study we have assessed the typical effects of physical cross-linking methods, namely ultraviolet (UV) light, dehydrothermal (DHT) treatment, and combinations of them on bovine articular CMD porous scaffolds with three different matrix concentrations (5%, 15% and 30%) to assess the relative strengths of each treatment. Our findings suggest that UV and UV–DHT treatments on 15% CMD scaffolds can yield architecturally optimal scaffolds for cartilage tissue engineering.

## 1. Introduction

Attempts to repair and regenerate impaired cartilage through cartilage tissue engineering have failed to meet the ultimate needs of most patients with articular cartilage injuries. Tissue engineering multi-factor strategies are still amongst promising approaches for repair of cartilage defects. Apart from the selection of suitable cells and growth factors, use of proper scaffolds with appropriate physiochemical structure [1] will favor biocompatibility and cell adhesion/proliferation. The scaffolds must possess suitable geometry and mechanical properties [2], high porosity and interconnectivity, stability and consistency of mechanical strength, and a proper surface micro-morphology [1]. Different three-dimensional (3D) constructs, such as complex branched helical micro-channels of micro-fluidic hydrogels, can provide good network structures [3]. The material from which the scaffold is fabricated plays a key role in chondroinduction. Various types of hydrogels, polymers, scaffolds and composites of different materials that can support cartilage matrix production have been tried [4–6]. It has been suggested that modified native extracellular matrix (ECM) may contain bioactive factors that can contribute to cell growth, migration, and differentiation. In general, the closer the material is to cartilage native matrix, the higher the probability of achieving a suitable engineered cartilage. For this reason, pure cartilage matrix derived (CMD) scaffolds have recently drawn attention as 3D culture of chondroinducable cells on cartilage matrix components seems logical, as they are expected to provide structural and biochemical signals at the same time [7–10].

Although CMD scaffolds provide numerous advantages for cartilage tissue engineering, it has been proven that the they exert weak compressive strength [11], leading to cell-mediated contraction and shrinkage of the construct [12]. As a result, the cells within the construct will have less access to the diffused nutrients and less room for multiplication and matrix production [13]. For this reason, various techniques have been developed to improve one or both aspects. Physical cross-linking strategies, such as ultra violet (UV) light and dehydrothermal (DHT) treatment for cartilage matrix components, have been found to enhance mechanical properties [14], while contradictory findings have been reported with chemical methods. Carbodiimide treatment was reported to retain the original dimensions of CMD scaffolds [8], and as well as glutaraldehyde treatment, it showed higher stiffing effects compared to DHT treatment [15]. The stiffer carbodiimide- or glutaraldehyde-treated CMD scaffolds showed higher cell attachment, proliferation and migration with perosteoblast cells compared to DHT-treated scaffolds [15]. A different study [8] reported on the inhibition of cell attachment and alterations in newly synthesized matrix composition in MSCs seeded on carbodiimide treated CMD scaffolds.

Scaffolds for cartilage tissue engineering are typically polymeric/biopolymeric 3D constructs meant to provide temporary physical, mechanical and biological support for chondrocytes and chondroinducible cells [16]. The main aim of the current study is to evaluate and compare the effects of different available physical treatments on architectural properties of bovine articular CMD porous scaffolds for cartilage tissue engineering applications. We first concentrate on physical structure (gross morphology, pore size, porosity and mechanical properties) of the CMD scaffolds as the main criteria, and then assess thermodynamic, infrared spectroscopy and biocompatibility data.

## 2. Materials and methods

### 2.1. CMD scaffold preparation

Bovine articular cartilage (BAC) was excised from both proximal and distal surfaces of metacarpophalangeal joints of calf hooves procured from a local slaughterhouse. BAC tissues from nine cow legs were lyophilized (FreeZone, Lanconco, USA) after weighing. The dry weights were measured using an electronic balance with a resolution of ± 0.0005 g (SHIMADZU AY220 Analytical Balance).

The rest of the cartilage tissue was pooled, minced, shattered and homogenized with a BIOSPEC Tissue TEAROR™ (#985370-395) in 1× phosphate buffered saline, 5 mMethylenediaminetetraacetic acid and 0.15 mMphenylmethylsulfonyl fluoride buffer solution at pH 7.4. The resulting slurry was centrifuged at 2000 rpm for 5 min, and the collected supernatant was further centrifuged at 6000 rpm for 5 min. The tissue slurry was permeabilized in 1% TritonX-100 (Fisher Scientific) prepared in 0.01 M Tris-HCl (Sigma # T5941) and left in a 4 °C refrigerator with gentle agitation for 12 h. After 12 h, the permeabilized slurry was centrifuged at 6000 rpm for 5 min, washed with PBS and incubated for 12 h at 37 °C in a hybridization oven with a low rotating speed in 50 U ml^−1^ DNAse I (Sigma # DN25), 1 U ml^−1^ RNAse A (Sigma) and 10 mM TrisHCl (Sigma # 88438) for decellularization. Finally, the slurry was washed twice with PBS and centrifuged at 6000 rpm for 5 min.

The scaffolds were produced by mixing the decellularized slurries with ultrapure water (UPW; conductivity = 0.055 ľ S cm^−1^ at 25 °C, total organic carbon value <1 ppb; membrapure Aquinity, Scientech, Taiwan) and placed in cylindrical teflon molds with a depth of 3 mm and a diameter of 7 mm. Scaffolds were prepared at 5%, 15% and 30% (w/v) concentrations, frozen at a constant temperature of –80 °C for 2 h in a freezer, then freeze-dried at −50 °C, 0.04 millibar for 16 h with no additional annealing steps, and post treated by one of the following: (A) no treatment (control); (B) exposed to UV light at an energy concentration of 8 J cm^−2^ for 90 min for each side (*λ* = 254 nm) (henceforth as ‘UV’); (C) dehydrothermal treatment, which means heating the scaffolds in a forced-air convection oven (Lab Companion OF-11E, China) at 120 °C for 24 h (henceforth as ‘DHT’); (D) UV followed by DHT treatment (‘UVDHT’); and (E) DHT treatment followed by UV treatment (‘DHTUV’). Although all dry scaffolds will be 100% composed of cartilage matrix, codes of the scaffolds are named as 5%, 15% and 30% CMD scaffolds based on the preparation concentration in the entire study.

### 2.2. CMD scaffold macro- and micro-morphologies

A digital camera (Canon PowerShot A570 IS) was used to capture cross-sectional images of the top view of the CMD scaffolds. ImageJ®, version 1.47v software was used for cross-sectional surface area (*A*_s_) measurement. Percentage of shrinkage was calculated using equation (1) for each type of scaffold with respect to the constant circular area of the mold (*A*_m_) with a diameter of 7 mm

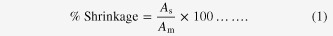



A field emission scanning electron microscope (FESEM) (Quanta™ 250 FEG—FEI) was used to assess micro-morphology, pore size and pore distribution. All scaffolds were gold-coated (at a thickness of around 450 Å) using a 150 rotary-pumped sputter coater (Quorum Technologies). ImageJ® software was used to calculate the pore sizes. Color thresholding in ImageJ® facilitates the detection of pore borders and enhances measurement accuracy. FESEM images of *n* = 3 samples from each group of scaffolds at a magnification of 100× were used for pore size measurement. Since all the identical samples from each concentration and treatment were uniform, the data of a representative scaffold from each group were analyzed for the pore size measurement. Each image was divided into nine virtual equal squares. Measurements were taken randomly from three of the squares. For each sample, *n* ≥ 50 bidirectional pore diameters were measured. The mean pore diameter was calculated from the average of the maximum and minimum diameters of a pore.

### 2.3. Porosity measurement

Scaffold porosity was measured through micro-volumetric modification of the liquid displacement method described elsewhere [17]. Briefly, changes in the hexane level in a glass pipette after immersing (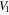) and removing (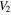) each scaffold (*n* ≥ 11 for each type of scaffold) were recorded by a digital camera and analyzed by ImageJ® software. The percentage of pore volume was calculated using equation (2):

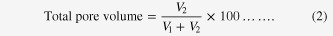



### 2.4. Thermogravimetric analysis (TGA)

Thermal stability of CMD scaffolds (*n* = 3 for each type of scaffold) was assessed using a thermogravimetric analyser (TA Instruments, Q500) at a constant heating rate of 10 °C min^−1^ in over a temperature range of 25–825 °C in a controlled nitrogen gas atmosphere.

### 2.5. Differential scanning calorimetry (DSC)

Melting temperature and cross-linking of CMD scaffolds (*n* = 3 for each type of scaffold)were analyzed using a Mettler DSC820 system (Mettler Toledo, UK) at a constant heating rate of 10 °C min^−1^ in over a temperature range of 25–100 °C in a controlled nitrogen gas atmosphere.

### 2.6. Fourier transform infrared (FTIR) spectroscopy

A FTIR spectrometer (Thermo Scientific® Nicolet iS10) was used to analyze the secondary structure of proteins within the CMD scaffold [18, 19]. Spectra were acquired from a 1.5 mm diameter sampling area (*n* = 3 for each type of scaffold and *n* = 3 measurements from three different spots per each sample) with a diamond crystal at a resolution of 4 cm^–1^ in the wave number region between 4000 and 650 cm^–1^.

### 2.7. Mechanical properties

Compressive strength and modulus of the scaffolds (*n* ≥ 7 for each group) with the dimensions of 6 mm in diameter and 2 mm height were measured in compression mode at a crosshead speed of 500 *μ*m min^−1^ using an Instron 5848 micro tester. The overall Young’s modulus was calculated from minimum (i.e. nearly at 0.7% strain) to maximum compressive strength (i.e. nearly at 95% strain).

### 2.8. Cell isolation and culture

Primary human dermal fibroblasts (HDFs) were isolated through the outgrowth method described elsewhere [20] from redundant skin from cosmetic-plastic surgery (excision from the gluteal region of a seven year old female). The clearance to conduct this study was provided by UMMC ethical approval (PPUM/MDU/300/04/03, 22 April 2011). Briefly, the dermis was dissected from the epidermis and the underlying fat tissue, and minced into pieces of ∼1 mm^2^. These were plated on 25 cm^2^ culture flasks pre-wetted with culture medium consisting of high-glucose Dulbecco’s modified Eagle’s medium (DMEM) (CellgroMediatech, USA) supplemented with 20% (v/v) fetal bovine serum (FBS)(HCL#SV30160.03 12, USA) and 1% penicillin–streptomycin–amphotericin B (Gibco 15240-062, USA). The cells were subcultured on 225 cm^2^ flasks (Corning C62-3001, USA)at 37 °C, 5% CO_2_ in the same culture medium, but supplemented with just 10% FBS and used at passage 4 for cell seeding. After harvesting and cell counting, 50 *μ*l aliquots of cell suspension containing 5 × 10^5^ cells (10^7^ ml^−1^) were seeded gently on top of each ethylene oxide sterilized scaffold (*n* ≥ 5 for each group) in a non-treated 48-well plate and incubated at 37 °C, 5% CO_2_ for 2 h, allowing the cells to diffuse into and attach to the scaffolds. A 1 ml culture medium consisting of high-glucose DMEM supplemented with 20% FBS, 1% penicillin–streptomycin–amphotricin B, L-ascorbic acid 2-phosphate (Sigma A8960, USA) (50 *μ*g ml^−1^), hydroxy L-proline (Sigma 5534, USA) (40 *μ*g ml^−1^), dexamethasone (Sigma D157) (10 nM), 1% HEPES (Sigma H4034), 1% non-essential amino acids (Sigma M7145, USA), 10% ITS + Premix (Corning 354352, USA), TGF-β3 (Sigma SRP3171, USA) (10 nM) was added later to each well. The media were changed every other day.

### 2.9. Cell viability and growth

At days 2, 7 and 14, HDF-seeded constructs (*n* = 1 for each group) were stained with LIVE/DEAD® Viability/Cytotoxicity Kit for mammalian cells (Invitrogen, UK) for confocal microscopy. Each construct was incubated for 45–60 min at 37 °C, 5% CO_2_ with 500 *μ*l PBS containing calcein AM (1:1000) to view live cells and ethidium homodimer (2.5:1000) to view dead cells. Imaging was performed using a confocal microscope (Leica TCS SP5 II, Germany).

Cell growth on scaffolds was also confirmed through total DNA measurement. At each mentioned time point (i.e., day 2, day 8 or day 15), the media were discarded. HDF seeded constructs (*n* ≥ 5) were washed gently with PBS, and subjected to overnight papain digestion, as described elsewhere [21]. DNA quantification was performed using Hoechst 33 258 (Sigma 861 405) [21]. Briefly, triplicates of 40 *μ*l of the digested construct solutions were transferred to a 96-well plate and topped up with 200 *μ*l Hoechst working solution (0.1 *μ*g ml^−1^). Serial dilution of calf thymus DNA (Sigma D4764) was used to create a DNA standard curve. Plate reading was undertaken at excitation: 355 and emission: 460 nm in a FLUOstar OPTIMA BMG LABTECH plate reader.

### 2.10. Statistical analysis

One-way, between-group analysis of variances (ANOVA) was conducted to explore the impact of concentration/treatment on shrinkage, pore size, porosity and Young’s modulus. A two-way ANOVA was performed to find the impact of treatment on DNA in UV and UVDHT treated 15% CMD scaffolds at three different time points. The significance value for Levene’s test of homogeneity of variances was assigned as *P* > 0.05, indicating that the assumption of homogeneity of variance has not been violated. Post-hoc comparisons using the Tukey HSD test were used to define the significant difference between the scaffolds with different concentration/treatments. A value of *P* < 0.05 was defined as significant.

## 3. Results and discussion

Weight measurements of wet and freeze-dried native BAC samples showed the average weight percentage of dry material in BAC to be 31 ± 3%, which is the maximum solid content of BAC samples.

### 3.1. Macro and micro morphologies of the CMD scaffolds

**Figure 1.**
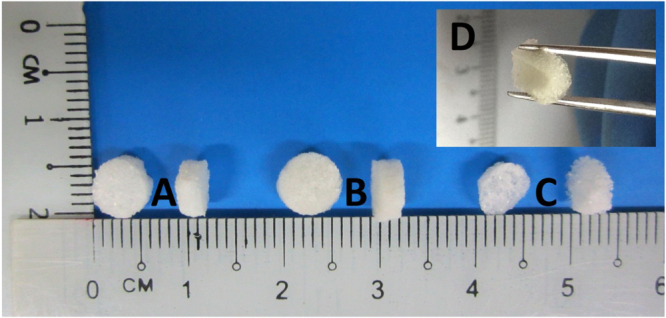
Photograph of representative non-treated (A) 30%, (B) 15% and (C) 5% CMD scaffolds. The inset image (D) depicts the spongy elastic nature of a 15% CMD scaffold.

Figure 1 shows the macroscopic morphologies and surface appearance of non-treated 30% (figure 1(A)), 15% (figure 1(B)), and 5% (figure 1(C)) CMD scaffolds. All samples show a highly porous spongy nature (figure 1(D)). While 15% and 30% CMD scaffolds retain their cylindrical shape, the 5% scaffolds look more deformed. All three concentration groups of scaffolds showed substantial changes in the size after freeze drying compared to their original mold sizes (shrinkage after lyophilization). Since there is a greater amount of water present in 5% CMD samples, the amount of total heat extracted from these samples during freeze-drying is much higher than those of 15% and 30% CMD scaffolds, leading to a higher level of shrinkage. However, differently treated samples displayed different rates of shrinkage, indicative of the role of treatment in the determination of shrinkage rate.

**Figure 2.**
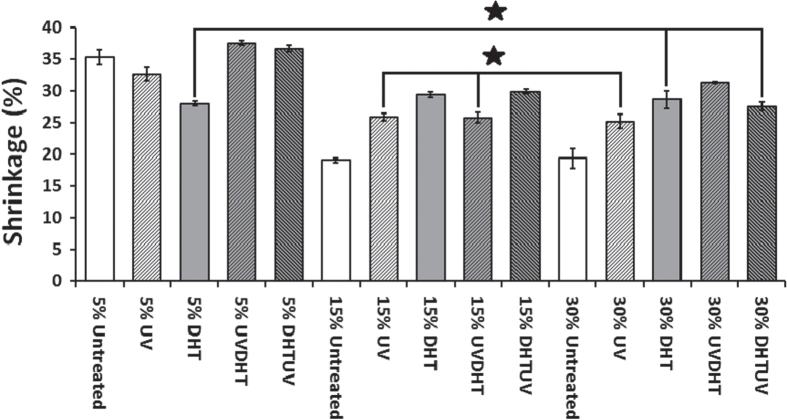
Percentage of shrinkage in CMD scaffolds with different concentrations and treatments (*n* ≥ 7 for each group). (∗: *P* > 0.05).

15% and 30% UV and 15% UVDHT scaffolds showed the lowest shrinkage (25.9 ± 1.5%, 25.9 ± 2%, and 25 ± 3%, respectively) among all treated CMD scaffolds as seen in figure 2. One-way ANOVA showed no significant difference in shrinkage between the three mentioned groups (*p* > 0.05).

**Figure 3.**
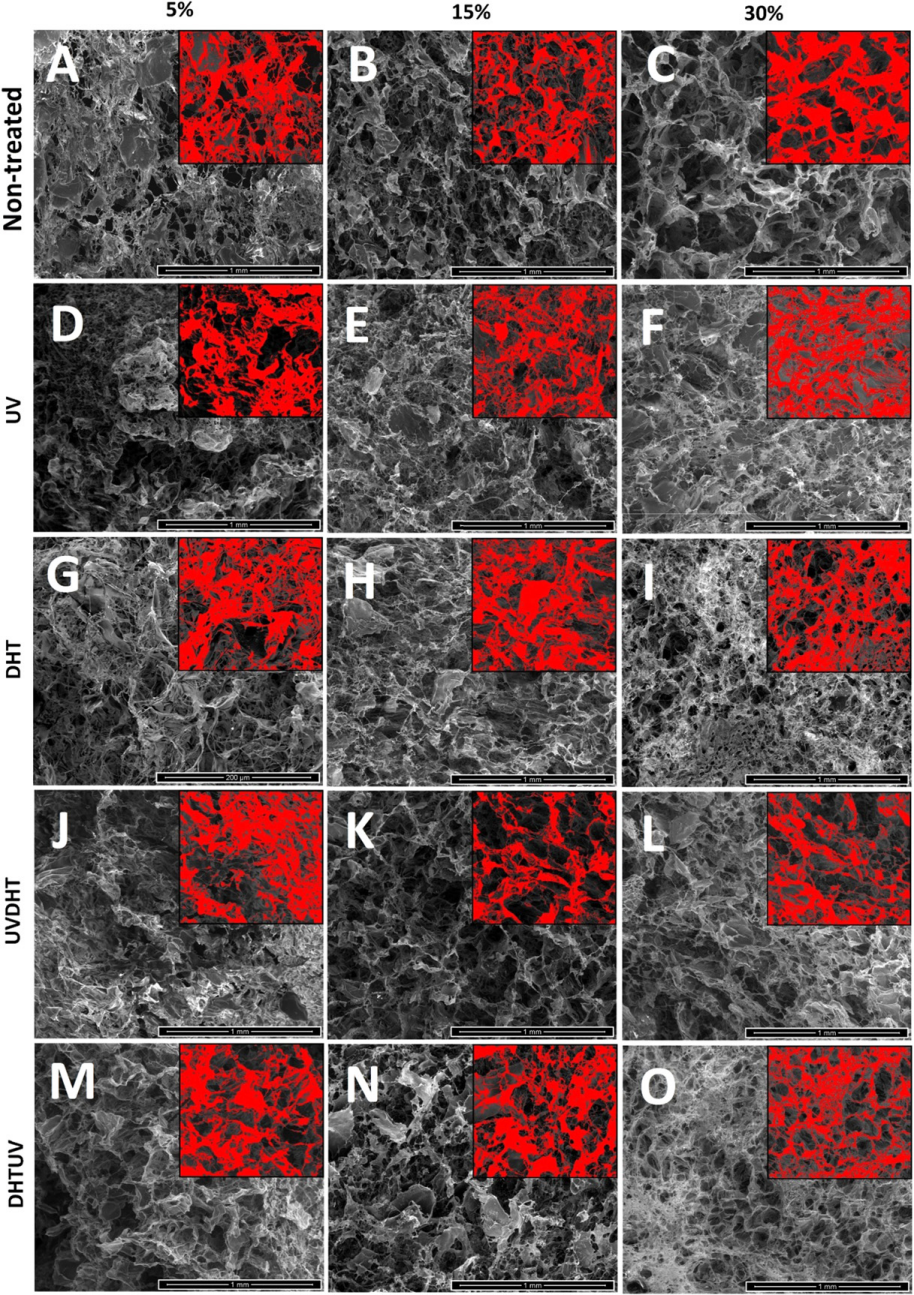
FESEM images of CDM scaffolds with different concentration and treatments. The scale bars represent 1 mm. Inset images with the same scale bars are representative of color thresholding through ImageJ® software used for pore size measurement.

**Figure 4.**
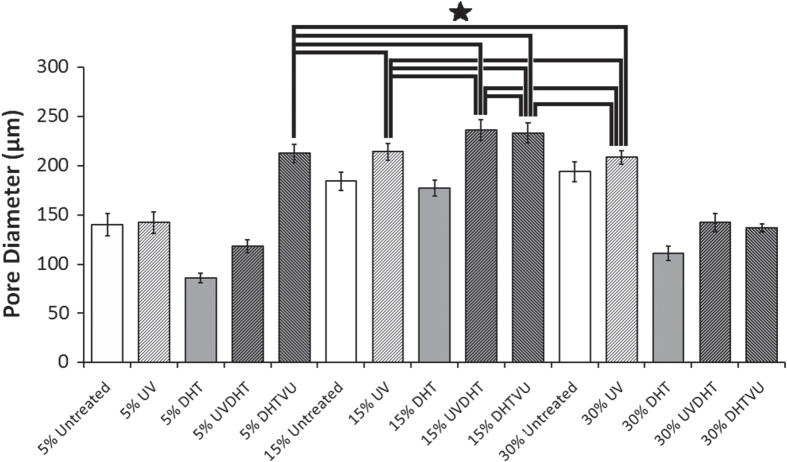
Pore sizes of non-treated and treated CMD scaffolds with different concentrations (*n* ≥ 50 for each group), calculated from FESEM images using ImageJ® software. (∗: *P* > 0.05).

FESEM images in figure 3 depict the uneven and irregularly shaped, highly interconnected pores among all 5%, 15% and 30% CMD scaffolds because of the freeze-drying scaffold fabrication method. Pore size analysis by ImageJ® showed an average pore diameter of 140 ± 60 *μ*m for non-treated 5% CMD scaffolds, and 184 ± 60 *μ*m and 194 ± 61 *μ*m for non-treated 15% and 30% scaffolds, respectively (figures 3(A)–(C)). The average pore size in non-treated 5% CMD scaffolds is significantly lower than that of non-treated 15% and 30% CMD scaffolds (figure 4). As it can be seen in the FESEM images of UV-treated CMD scaffolds (figures 3(D)–(F)), UV treatment did not affect the pore size of 5% scaffolds, but did show moderate and mild increases in the pore sizes of 15% and 30% scaffolds, respectively. The correlation between the shrinkage and pore sizes of the CMD scaffolds was small (*R*^2^ = 0.097). Shrinkage of the scaffolds during freeze-drying is not necessarily associated with a change in pore size, and pore size is determined by the rate of cooling and annealing within the lyophilization process [22]. However, the increment in pore size of 15% and 30% scaffolds seems to be coincident with the over 5% increase in shrinkage in the 15% and 30% scaffolds, suggests that the degrading effect of UV on loose bands simultaneously results in smaller scaffolds with bigger pore sizes.

Image analysis of DHT treated scaffolds (figures 3(G)–(I)) shows a significant drop in the pore size of 5% and 30% scaffolds, compared with a light decrease in 15% scaffolds with respect to their original non-treated pore sizes.

As it can be seen in figures 3(J)–(L), UVDHT treated 15% and 30% scaffolds show the biggest pore sizes among all other samples, while the pore sizes of 5% UVDHT are even smaller than the non-treated 5% scaffolds. FESEM images of DHTUV samples (figures 3(M)–(O)) indicate big pore sizes for 15% samples, which are almost the size of those for the 15% UVDHT, but a huge decrease in the pore sizes in 30% DHTUV scaffolds. Interestingly, 5% DHTUV samples showed the biggest pore sizes among all other 5% scaffolds with different treatments.

Figure 4 shows the average pore sizes of CMD scaffolds with different concentrations and treatments (*n* ≥ 50 for each group) derived from FESEM images using ImageJ® software. The CMD scaffolds with 5% DHTUV, 15% UV, UVDHT and DHTUV, and 30% UV treatments showed the biggest mean pore diameters among all the different scaffolds. One-way ANOVA between the five mentioned groups revealed no significant difference between them (*P* > 0.05).

**Figure 5.**
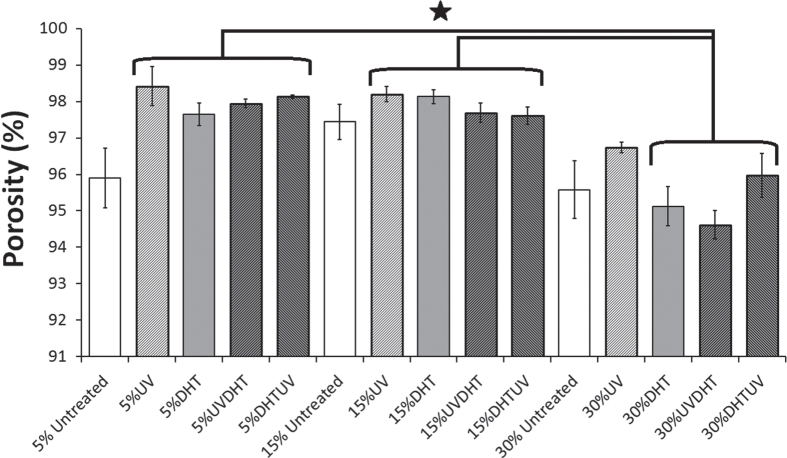
Percentage of porosity of different CMD scaffolds with various cartilage matrix concentrations and different treatments (*n* ≥ 11 for each group) through microvolumetric modification of Archimedes liquid displacement method. (∗: *P* < 0.05).

Porosity measurement by a modified microvolumetric Archimedes method showed an average porosity between 94.6% and 98.4% among all different CMD scaffolds (figure 5). Considering the fact that the biggest difference between the porosities is still less than 4% among all the different groups, it can be noticed that all scaffolds could still be assumed as highly porous and the difference is rather significant, but the resultant increase in surface area can actually be beneficial for cell attachment and migration.

**Figure 6.**
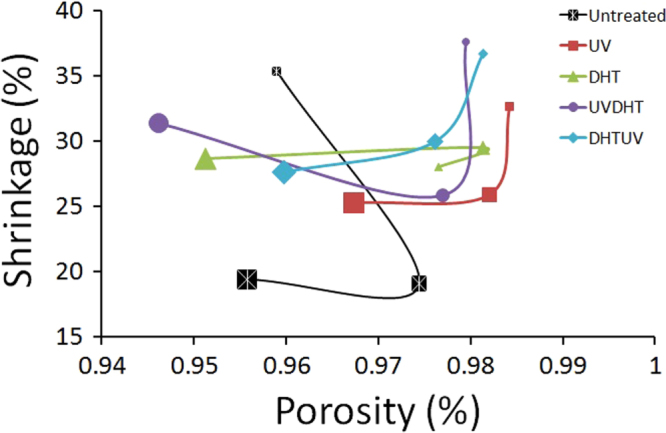
Correlation between shrinkage and porosity among CMD scaffolds with different concentrations and treatments. Markers with small, middle and big sizes indicate for 5%, 15% and 30% cartilage matrix concentrations, respectively.

Plotting the shrinkage against the porosity shows that increasing the concentration of CMD from 5% to 15% does not change the porosity of all treated and untreated scaffolds significantly (*P* > 0.05), while it significantly decreases the shrinkage in all treatments (*P* < 0.05) except for DHT (*P* > 0.05). However, increasing the CMD concentration from 15% to 30% does not have significant changes in shrinkage of all treatments unless for DHT group, while the differences in porosity of different treatments are significant (*P* < 0.05). This result suggests that 15% CMD concentration is a critical value beyond which significant changes in porosity and shrinkage occur (figure 6).

All 5% and 15% treated scaffolds were at 98%, while the highest porosity among the 30% group was in UV-treated scaffolds at 96.7 ± 0.5%. This result indicates that when the scaffolds were shrinking, the shape and size were changed significantly without much change in the total number of pores, resulting in a similar porosity of over 94% for all specimens. Furthermore, the higher porosity in a few treated 5% CMD scaffolds is indicative of breaking of the surface (as revealed in thresholding inset images in figure 3 for 5% scaffolds) as a result of increased open porosity. Since 30% CMD concentration is more than a critical value, which is in this pure water media 15% CMD, the total number of pores also decreases with a greater amount compared with 5 or 15% CMD scaffolds.

Pluralization of shrinkage, pore size and porosity data as major criteria in scaffold engineering suggests that although 5% and 15% DHTUV groups show big pore sizes, but simultaneously show high percentages of shrinkage, they are disqualified for selection as good scaffolds.

On the other hand, the 15% and 30% UV and also 15% UVDHT groups show the lowest shrinkages, proportionally biggest pore sizes and high porosities, and hence all three groups can be scaffolds of choice from the view of architecture.

Considering all the above characteristics, including shrinkage, pore size and porosity of the entire CMD scaffolds, and to confirm our selection, the other data of all 15% CMD scaffolds was assessed and characterized further.

### 3.2. Compressive tests

**Figure 7.**
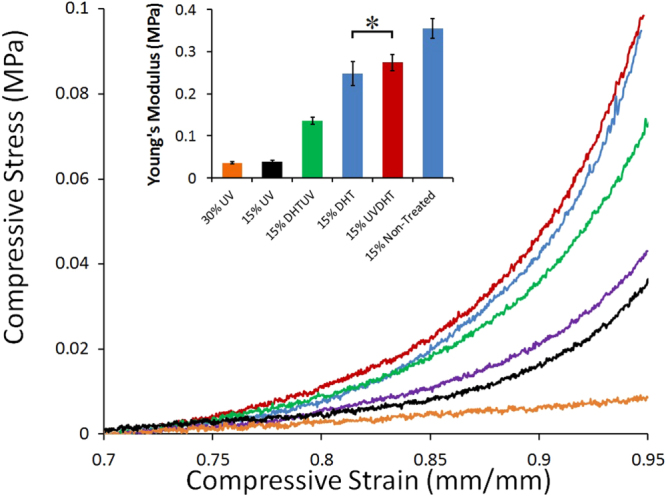
The compressive stress/strain representative curves of untreated and treated 15% and 30% UV-treated CMD scaffolds. The inset graph compares Young’s modulus (*n* = 7) of differently treated 15% CMD scaffolds. (∗: *P* > 0.05).

Figure 7 shows the results of mechanical testing in compression mode for all treatments of 15% along with 30% UV-treated CDM scaffolds. UV and UVDHT treated scaffolds showed the highest compressive modulus (*E*_c_) among other scaffolds, while one-way ANOVA showed that Young’s modulus of DHT treated 15% scaffolds was not significantly different from that of UVDHT scaffolds (*p* = 0.874). On the other hand, the 30% UV-treated scaffolds exerted weak compressive strength and low Young’s modulus. Considering the maximum physiologic load applied on knee joint cartilage, which is said to be 0.84–3.0 MPa for an average person, the Young’s moduli measured for 15% UVDHT- and UV-treated CMD scaffolds (0.274 ± 0.05 MPa and 0.355 ± 0.06 MPa, respectively) falls between the range of 10–42% of compression moduli values of natural cartilage [16]. The Young’s moduli for 15% UVDHT and UV-treated CMD scaffold are significantly higher than untreated 15% CMD scaffolds and significantly higher than the compressive moduli reported for potential scaffold substitutes such as 1.5% and 2% agarose (9.0 ± 0.3 kPa and 76 ± 5 kPa, respectively) and mechanically enhanced agarose/PEG-dimethacrylate (51 ± 3 kPa–93 ± 4 kPa) hydrogels [23].

### 3.3. Surface morphology

**Figure 8.**
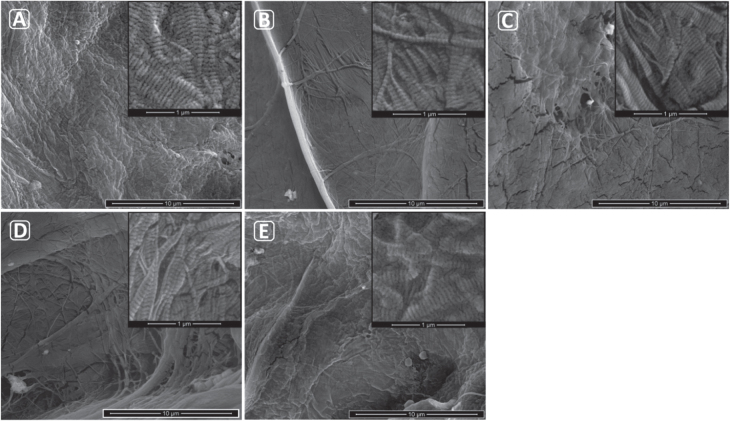
FESEM images of fibrous surface morphology of (A) non-treated, (B) UV, (C) DHT, (D) UVDHT and (E) DHTUV 15% CMD scaffolds. The scale bars represent 10 *μ*m (for insets, 1 *μ*m).

Figure 8 illustrates the surface topography changes in 15% CMD scaffolds after different cross-linking treatments, compared to the non-treated sample at higher magnification. The typical nano-structure of collagen fibrils, with their bands, is revealed in all samples under the very high magnification inset images in figure 8. A ramous surface morphology is seen in all treated samples, while non-treated material shows no signs of dehiscence. This indicates that more cross-linking between the collagen polymer chains in the fibrils has occurred due to the different physical treatment. The DHT sample shows more surface cracks compared to other materials (figure 8(C)). On the other hand, UV- and DHTUV-treated materials (figures 8(B) and (E)) show the fewest cracks. In addition, the UV-treated scaffolds show many network structures indicative of maximum cross-linking, and the UVDHT-treated scaffolds are moderately ramous. Considering these results, UV and UVDHT appear to have the more optimal surface morphologies, compared to the other treated or non-treated scaffolds.

### 3.4. TGA

**Figure 9.**
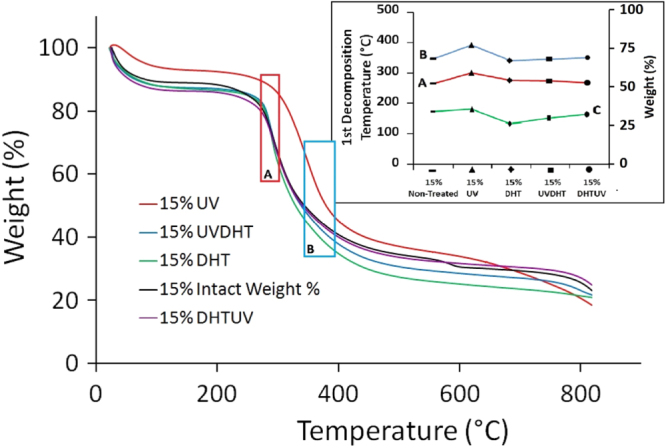
TGA of all the 15% CMD scaffolds in nitrogen gas atmosphere at heating rate of 10 °C min^−1^, (A) onset and (B) offset temperatures of 1st decomposition, and (C) weight residue (%) after 1st decomposition of differently treated 15% CMD scaffolds in TGA.

All CMD materials show two main decomposition temperatures according to the two major weight losses (figure 9). The first weight loss (10–16 wt %) appears at around 265 °C and is due to adsorbed moisture, adsorbed water and collagen present in the material. The next decomposition (56–66 wt %) occurs at almost 720 °C and represents the inorganic components in cartilage.

Increases in thermal stability, which are measured in terms of the first decomposition temperature, is a result of and proportional to formation of cross-linked structures [24]. The 15% UV scaffold group showed the highest thermal stability of all treatments. The onset and offset temperatures of the first decomposition of 15% UV-treated sample computed at 300.85 and 393.7 °C, respectively, which is noticeably higher than those of other treatments (see the inset in figure 9). The average weight residue after the first decomposition is 36 ± 4% regardless of treatment for all 15% samples (see the inset in figure 9).

### 3.5. DSC

**Figure 10.**
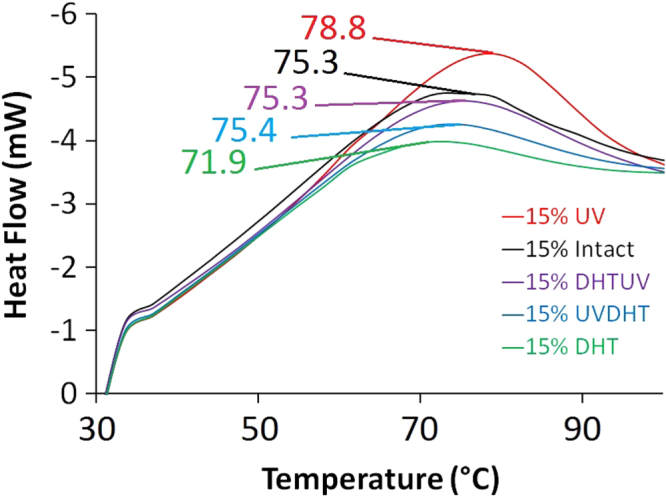
DSC analysis of 15% CMD scaffolds with different treatments indicating the melting temperature of each treatment.

Figure 10 shows the DSC results and indicates melting points (*T*_m_, in °C) of the 15% CMD scaffolds with different treatments. The 15% UV-treated scaffold shows a significantly higher melting temperature compared to the other treated and non-treated scaffolds, suggesting that it may have better mechanical properties, which has already been shown in a mechanical study. A higher melting point occurs due to the more cross-linking present in the 15% UV-treated scaffolds than in other non-treated or treated scaffolds. The cross-links may have been damaged by thermal energy in other treated scaffolds or by other treatments.

### 3.6. FTIR

**Figure 11.**
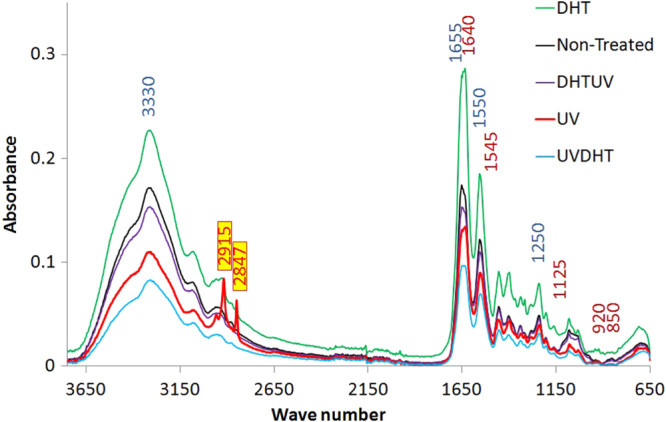
Typical FTIR absorption spectra of 15% CDM material with different treatments. Wave numbers defined in blue and dark red are representative for collagen and proteoglycans respectively. The two sharp peaks at 2847 and 2915 on 15% UV are representative for alkane groups formed during UV treatment.

The FTIR spectra of 15% CDM scaffolds with different treatments are shown in figure 11. Articular cartilage extracellular matrix derived scaffolds mainly consist of collagen II and proteoglycans. amide I (C = O stretch), amide II (N–H stretch, N–H bend combination), amide III (C–N stretch, N–H bend, C–C stretch) and amide A (N = H stretch) at wave numbers 1655, 1550, 1250 and 3330 cm^−1^, respectively, are the representative bonds of collagen, while sulfate stretch (C–O–S stretch) occurred at 1125-920 and 850 cm^−1^, amide I (C = O stretch) at 1640 cm^−1^ and amide II (N–H stretch, N–H bend combination) at 1545 cm^−1^ are characteristic of proteoglycans [19, 25–28]. All the characteristic FTIR peaks, including amides I, II, III and A, as well as sulfate groups for collagen and proteoglycans, are shown in figure 11.

**Figure 12.**
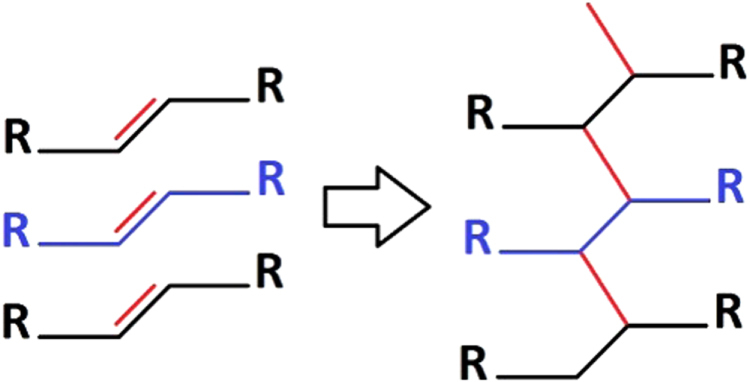
Alkene to alkane shifts will create new cross-linking bonds.

The two sharp peaks at 2847 and 2915 cm^−1^ wave numbers on 15% UV group spectrum are due to new alkane group formation from alkene groups during UV cross-linking treatment (figure 12). The broken bonds from the alkenes in turn help the alkanes to make more cross-links with the other chains of polymer collagens, as shown in figure 11. The more cross-links through UV in 15% CMD scaffolds would restrain the scaffold structure during cell culture study better than other treated scaffolds.

Considering the sensitivity of the amide III band at 1235 cm^−1^ to native collagen tertiary structure and C–H deformation related insensitivity of the absorbance at 1450 cm^−1^, the 1235/1450 cm^−1^ ratio can help in quantification of degradation [29, 30]. The ratio was 1.022 in untreated 15% CMD scaffolds and showed a decrease after treatment with UV (1.011) and UVDHT (1.008), indicating higher cross-linkage in initially UV-treated samples, while increasing in DHT (1.030) and DHTUV (1.023) treated scaffolds resulting from a partial degradation in samples first treated with DHT.

Our FTIR findings show that the effectiveness of physical treatment of CMD constructs is highly dependent on the concentration of CMD content in the construct. At low CMD concentrations (e.g., 5% CMD in our case), UV treatment causes more cross-linking, reconfirming the findings of Bellincampi and Dunn [31]. When the CMD concentration is increased to 15%, the DHT treatment forms a higher cross-linking, while the UV treatment has more denaturation effects, which replicate the findings of a study on collagen fibers conducted by Weadock *et al* [32]. At the same time, alkene to alkane transitions provide supportive cross-linkings.

### 3.7. Cell growth and viability

**Figure 13.**
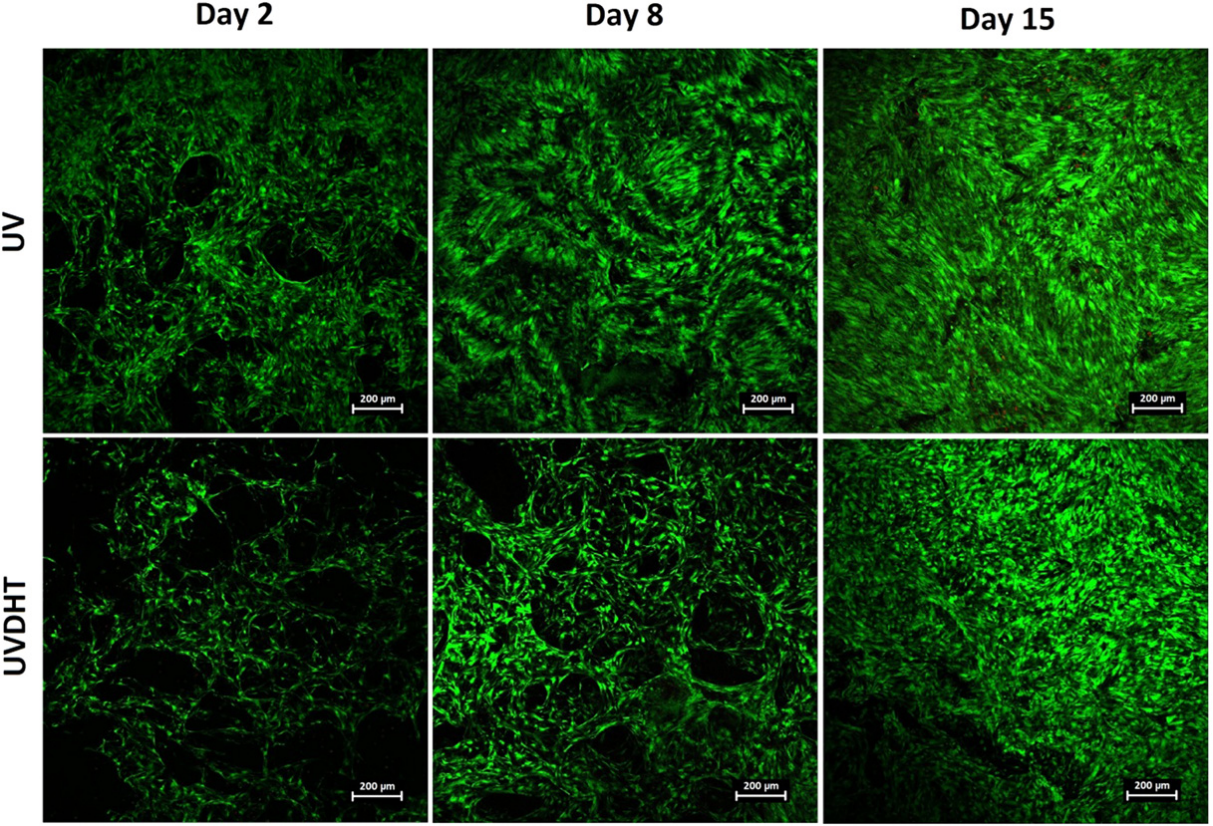
Confocal microscopy images of UV- and UVDHT-treated 15% porous scaffolds at different time points after seeding HDF cells. The scale bars represent 200 *μ*m.

Confocal images of HDF seeded 15% UV- and UVDHT-treated constructs at three different time points (days 2, 8 and 15) are presented in figure 13. The porous nature of scaffolds can be clearly seen from the style of cells attaching to the constructs, especially in the day 2 and day 8 images. Progressive growth of HDF cells on both types of UV- and UVDHT-treated scaffolds can be clearly traced in these images, showing the cells growing on the surfaces and filling in the pores.

**Figure 14.**
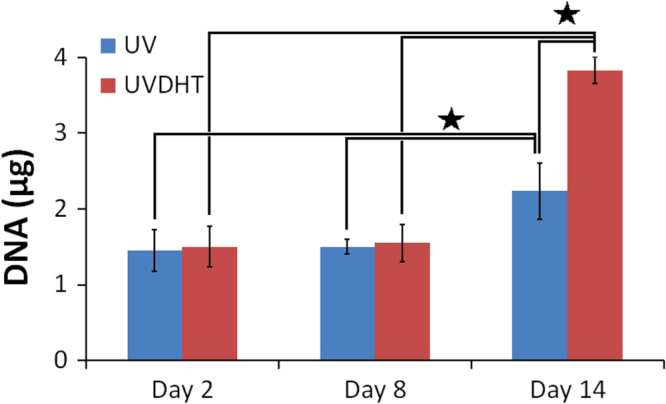
Total DNA measurement after subtraction of DNA content in unseeded samples. DNA quantification has been performed through the Hoechst method after papain digestion of HDF seeded 15% UV and UVDHT scaffolds (*n* ≥ 5 in each group) at three sequential weekly time points. (∗: *P* ≤ 0.05).

DNA quantification showed a considerable amount of DNA in unseeded scaffolds (4.0 ± 0.6 and 3.6 ± 0.6 *μ*g for UV and UVDHT groups, respectively), which is due to incomplete decellularization and the DNA wash-out process. Considering the seeding density of 5 × 10^5^ cells seeded on each scaffold containing an average total DNA content of 3.25 *μ*g (≈6.5 pg/cell) [33, 34] and the blank, corrected DNA content at the 2nd day after seeding. It can be inferred that nearly half the seeded cells have attached to the scaffold surfaces by day 2. The DNA content did not change significantly during the first week after culture (*P* > 0.05) but showed a significant increase in both groups by the end of the 2nd week, with a significantly higher trend in the UVDHT group (*P* ≤ 0.05) (figure 14). This increase in total DNA during the two-week time span indicates the growth of cells on both 15% UV- and UVDHT-treated CMD scaffolds.

## 4. Conclusions

ECM-based strategies are already established in tissue engineering [35]. Native cartilage ECM-derived scaffolds have been shown to support chondrogenesis [36]. Also, the effects of physical (UV and DHT) and chemical (carbodiimide) cross-linking treatments on chondrogenic differentiation and cell-mediated contraction of porous CMD scaffolds have already been studied [8]. However, the purpose of the current study was quantitative assessment of changes in the architectural and mechanical properties of physically cross-linked CMD scaffolds.

Considering other studies done on 3% [37] and 10% [38] CMD scaffolds, and according to the proportional weight of dry material content in native fresh BAC, we produced bovine CMD scaffolds with three different concentrations of CMD material: 5%, 15% and 30% concentrations.

The scaffolds underwent one of the five physical treatment conditions for cross-linking (non-treated, UV, DHT, UVDHT and DHTUV). Remarkable deformation and shrinkage after treatment and small pore size were the major criteria to eliminate the 5% scaffold group. UV- and UVDHT-treated 15% scaffolds, as well as UV-treated 30% scaffolds, showed the biggest pore sizes amongst all other samples. Although UV-treated 30% scaffolds have the same concentration of dry material as the native cartilage, they showed a lower porosity, a relatively higher shrinkage, and a very low compressive strength compared to UV- and UVDHT-treated 15% scaffolds. Structural stability, sufficient surface area for cell attachment, adequate space for diffusion of nutrients, cell migration and matrix deposition have been accounted as beneficial features of scaffolds with 95–97% porosity [39]. UV- and UVDHT-treated 15% CMD scaffolds showed high porosity rates (≈98%) and high Young’s moduli. The compressive moduli of these scaffolds appeared to be higher than pure and enhanced agarose and agarose/PEG [23], cell-seeded porous scaffolds derived from native porcine articular cartilage [36], and close to PEG hydrogels [40] as potential alternative constructs for cartilage tissue engineering.

While both UV- and UVDHT-treated 15% scaffolds showed high pore sizes, low shrinkages and high porosities, UV-treated 15% scaffolds showed a higher compressive strength, better thermal stability in TGA and DSC analyses, as well as newly formed alkane peaks in FTIR, which are obviously indicative of a higher degree of cross-linking.

As confirmed by FTIR study, cross-linking changes the mechanical strength of the scaffold through breaking the unsaturated bonds between the polymer chains and converting them to primary bonds. The breaking of unsaturated bonds and consequent transformation into the primary bonds are mainly responsible to change the porosity in the CMD scaffolds as revealed in the SEM micrographs. The surface morphology of UV-treated 15% scaffolds also showed an optimal intertwined collagenous surface that favours better cell attachment. UV- and UVDHT-treated 15% scaffolds also showed good biocompatibility in terms of cell attachment and proliferation.

DHT treatment has been reported to have beneficial effects in terms of providing bigger pore volume favouring cell attachment, proliferation, matrix production and cell–matrix interactions [8]. DHT treatment was also reported to increase the mechanical properties of collagen–glycosaminoglycan scaffolds [14]. Our findings indicate that although the compressive profile of DHT treated 15% CMD scaffolds as well as its porosity are as good as UVDHT-treated CMD scaffolds, DHT treatment results in higher shrinkage and smaller pore sizes at 15% cartilage matrix concentration compared to UV and UVDHT treatments. UV and UVDHT treatments on 15% CMD scaffolds can yield stiffer CMD scaffolds with low shrinkage rates that simultaneously possess optimal microstructure and biocompatibility. Our study covered 5%, 15% and 30% CMD scaffolds and, based upon data obtained, suggests that 15% CMD concentration is a critical value beyond which significant changes in porosity and shrinkage are happening with physical treatment.

Therefore, simple, feasible and cost effective UV and UVDHT treatments on 15% CMD scaffolds are promising candidates to provide the desired architecture while preserving bioactive factors, both of which are major concerns in the tissue engineering of scaffolds.

Future studies are required to elucidate the particular chondroinductive capabilities of physically treated CMD scaffolds, and to further characterise the results of the current study.
